# Influence of residual buccal bone thickness in dehiscence defects on osseointegrated dental implants in healed sites: an experimental *in vivo* study

**DOI:** 10.1590/1807-3107bor-2025.vol39.079

**Published:** 2025-09-08

**Authors:** Guilherme Carlos Beiruth FREIRE, Patricia Furtado GONÇALVES, Suzana Peres PIMENTEL, Francisco Humberto NOCITI, Márcio Zafalon CASATI, Bruno César de Vasconcelos GURGEL

**Affiliations:** (a) Universidade Federal do Rio Grande do Norte -UFRN, Department of Dentistry, Natal, RN, Brazil.; (b) Universidade Federal dos Vales do Jequitinhonha e Mucury - UFVJM, Department of Dentistry, Diamantina, MG, Brazil.; (c) Universidade Paulista - UNIP, School of Dentistry, Dental Research Division, São Paulo, SP, Brazil.; (d) Universidade Estadual de Campinas – Unicamp, Piracicaba School of Dentistry, Department of Prosthesis and Periodontology, Piracicaba, SP, Brazil.

**Keywords:** Dental Implants, Bone Regeneration

## Abstract

This study aimed to histomorphometrically evaluate the effect of guided bone regeneration (GBR) and two implant surfaces on the thickness and height of newly formed bone in dehiscence defects around titanium implants. Three premolars and the first bilateral molar were extracted from ten adult mongrel dogs, and 40 buccal bone dehiscences measuring 5 mm in height and 4 mm in width were created using a University of North Carolina (UNC) periodontal probe to confirm the dimensions. Forty implants were randomly assigned to one of four groups: oxidized implant surfaces (OIS, n = 10), turned/machined implant surfaces (TIS, n = 10), OIS + GBR (n = 10), and TIS + GBR (n = 10). After 3 months, the dogs were euthanized, and the blocks containing the implants and adjacent bone were processed for non-decalcified histological analysis. Statistical analysis was performed using two-way ANOVA and the Pearson correlation (p = 0.05). The results showed that GBR significantly influenced both the vertical (height) and horizontal (thickness) dimensions of the newly formed bone (p < 0.001). Strong positive correlations were observed between the thickness and height of newly formed bone at the base of the defect, as well as between the thickness of the bone at the base of the defect and the thickness of newly formed bone in the central region of the defect. No significant correlations were found between implant surface type and bone formation. Bone regeneration occurred in both the vertical and horizontal directions, with greater bone growth in GBR-treated groups, irrespective of implant surface type (oxidized or turned).

## Introduction

Bone defects around dental implants occur frequently, even when implants are correctly positioned within the jaws.^1^ Dehiscence or fenestration defects can compromise implant rehabilitation and increase the risk of failure.^2^ In such cases, guided bone regeneration (GBR) has demonstrated effective clinical outcomes.^3,4^ GBR is a widely used and predictable technique that facilitates the formation of new bone over the exposed implant surface.^1,3,5,6^

The use of a membrane acts as a barrier that stabilizes the blood clot, prevents soft tissue invasion into the bone defect, facilitates the migration of bone-forming cells, and consequently promotes bone regeneration.^7,8^ According to Dahlin et al.,^2^ expanded polytetrafluoroethylene (ePTFE) membranes placed over fenestrations around dental implants successfully promoted localized bone formation. Achieving high implant survival rates and maintaining stable peri-implant tissues with this approach depends on various factors, including defect size.^1,4^ Furthermore, previous studies have suggested that implant surface modification enhanced cell adhesion, osteoconduction, and bone formation during the healing process.^9,10^Yang et al.^11^ reported that treated implant surfaces achieved superior osseointegration compared to smooth-surface implants. However, other studies indicate that smooth surfaces are easier to decontaminate, exhibit reduced biofilm accumulation, and are associated with lower inflammatory responses.^12^

Vertical and horizontal bone formation is critical for implant anchorage and proper distribution of functional forces.^11^ However, residual buccal bone thickness in dehiscence defects also affects bone healing after implant placement in healed sites. Additionally, the combined effects of different implant surface treatments and GBR using non-resorbable membranes on bone formation require further investigation. Therefore, this experimental study in dogs aimed to address two key questions through histometric analysis: (a) Does the use of a non-resorbable membrane influence the height and thickness of newly formed bone, irrespective of implant surface treatment? and (b) How do different implant surface treatments affect bone formation with and without GBR in dehiscence defects?

## Methods

### Animal model

Ten healthy mongrel dogs, each weighing approximately 25 kg, were included in this study. The research was approved by the Animal Research Ethics Committee of the University of Campinas (protocol 555-I), and adhered to ARRIVE (Animal Research: Reporting of In Vivo Experiments) Guidelines.^13^

### Surgical procedure and dental extraction

The dogs were sedated with dihydrothiazine hydrochloride (1.5 ml/10kg). General anesthesia was induced via intravenous injection of 2.5% thiopental solution (1.0 ml/kg), after sedation with intramuscular dihydrothiazine hydrochloride at a dose of 5 ml/10kg, supplemented with local administration of 2% lidocaine (1:100,000). The procedure was performed by a veterinary surgeon, who also monitored the entire process. A full-thickness flap was raised, and the second and third bilateral premolars were extracted.

**Figure 1:**
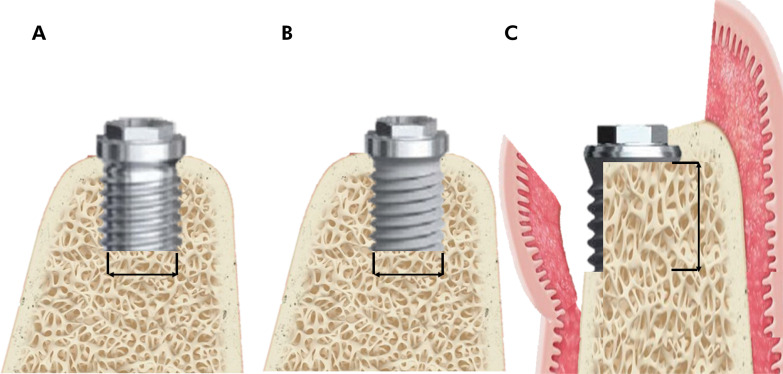
Schematic illustration of the surgically created dehiscence defect. (a) Front view of the bone defect with a turned surface implant. (b) Front view of the bone defect with an oxidized surface implant. (c) Lateral view of the center of the bone defect. Lines indicate defect height and width.

After three months of bone healing, the mandibles were radiographed, and the sites were prepared for bilateral implant placement. Before implant installation, 40 standardized, rectangular dehiscence-type bone defects were created, each measuring 5 mm in height and 4 mm in width, with four defects per dog. The defects were prepared using a high-speed cylindrical drill under saline irrigation, with a University of North Carolina (UNC) periodontal probe used to ensure dimensional accuracy. The study design included four experimental groups, combining two variables: implant surface treatment and membrane use. After site preparation, two screw-shaped, pure cylindrical titanium implants with an oxidized titanium surface (OIS) (TiUnite MKIII, Branemark System, Nobel Biocare, Göteborg, Sweden)—a microrough surface achieved through anodization—and two machined titanium surface (TIS) implants (MKIII, Branemark System, Nobel Biocare) were manually inserted at the bone level on each side of the mandible, following the manufacturer’s recommendations. All implants measured 8.5 mm in length and 4 mm in diameter. The total number of external hex implants placed was 40 (OIS, n = 10; TIS, n = 10; OIS + GBR, n = 10; TIS + GBR, n = 10) (Figures 1a, 1b, and 1c).

### Defect treatment

**Figure 2:**
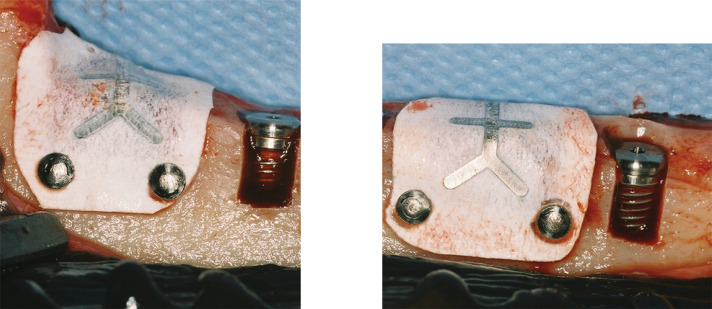
Clinical appearance after implant and membrane placement: (a) oxidized surface implant; (b) turned surface implant.

Immediately after implant placement, Y-shaped, non-resorbable ePTFE membranes (Gore-Tex, TR4Y, Flagstaff, USA) were randomly placed over two implants per site—one with a treated surface and one with a turned surface—both exhibiting dehiscence defects. The membranes were secured using non-resorbable metal pins (Figures 2a and 2b). The remaining two implants, one with each surface treatment, were left without GBR, serving as controls for the membrane variable while maintaining the surface treatment comparison. The mucoperiosteal flaps were repositioned and sutured using non-resorbable ePTFE sutures, which were removed 15 days postoperatively. For oral hygiene, 0.12% chlorhexidine spray was applied once daily for one month. Additionally, biofilm and calculus removal was performed monthly as needed through scaling, root planing, and prophylaxis.

### Histology and histomorphometry

The animals were euthanized three months after implant placement and defect treatment by deepening anesthesia, followed by a lethal injection of 19.1% potassium chloride. The mandibles were removed, dissected, and sectioned to obtain blocks containing the experimental samples. These were immersed in a 4% buffered formalin for fixation. Non-decalcified buccolingual sections (70–85 mm thick) were prepared from the middle portion of the defect, parallel to the implant axis, as previously described.^14^

**Figure 3:**
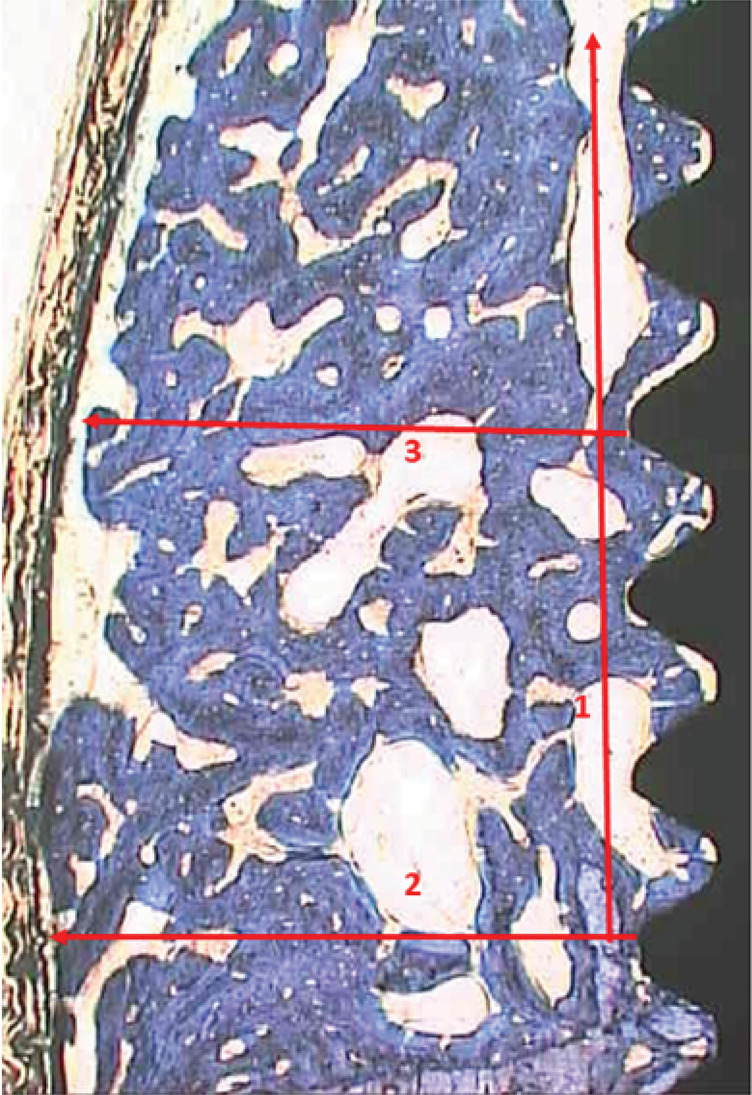
Histometric measurements of vertical bone formation: (1) horizontal measurement at the base of the bone defect; (2) horizontal measurement at the center of the bone defect; (3) representative image of an oxidized implant surface with GBR (original magnification: 3.12x).

The sections were stained with 1% toluidine blue, and one representative section from the middle portion of each implant was selected for histometric analysis. Histometric measurements were performed using ImageJ software by a blinded, calibrated examiner (kappa = 0.75). Three measurements were recorded, as illustrated in Figure 3:

- Height relative to the base of the bone defect (HD): vertical distance (mm) from the uppermost part of the first thread to the uppermost part of the sixth thread.
- Thickness relative to the base of the bone defect (TDb): horizontal distance (mm) from the tip of the sixth thread to the outermost region in contact with the peri-implant mucosa or membrane.
- Thickness relative to the central region of the bone defect (TDc): horizontal distance (mm) from the tip of the thread in the central portion of the dehiscence defect to the outermost portion of the bone in contact with the peri-implant mucosa or membrane.

### Statistical analysis

A randomized block design was used in this study, since each dog received all the treatments. Statistical analyses of the datasets were performed using IBM SPSS Statistics 22.0 (IBM Corp, Chicago, USA). Mean values and standard deviations (SDs) were calculated for all parameters across the experimental groups (OIS, TIS, OIS + GBR, and TIS + GBR). The Shapiro-Wilk test was used to assess data normality. A two-way ANOVA was performed to detect differences between groups for each of three measurements (HD, TDb, TDc) in independent samples, comparing the two implant surfaces and the presence or absence of the membrane. The Pearson correlation was used to assess relationships between height and thickness variables at the base and center of the defect. A significance level of 5% (α = 0.05) was set for all analyses.

## Results

No animals were excluded from the study, and no adverse events were recorded. All 40 implants and the membranes remained stable throughout the research period, with no signs of inflammation or exposure. Two-way ANOVA revealed no significant effect of implant surface on bone formation in any of the three analyzed regions. However, GBR significantly influenced bone formation, affecting:

- Height of newly formed bone (p = 0.008)
- Bone thickness relative to the base of the defect (p < 0.001)
- Bone thickness relative to the central portion of the defect (p < 0.001)

**Figure 4:**
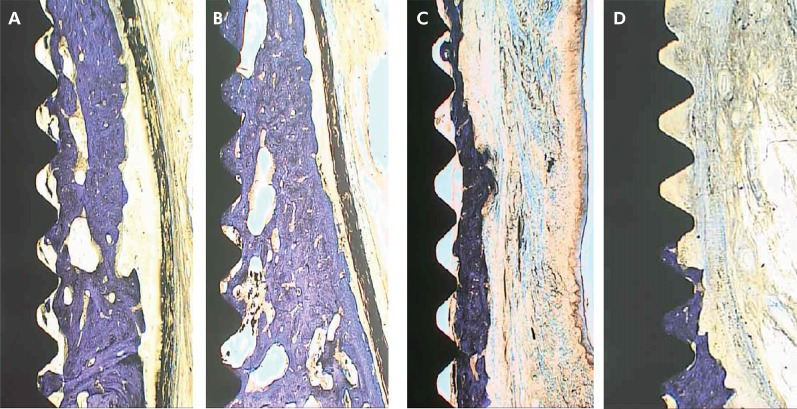
Representative histological images from each experimental group: (a) TIS + GBR; (b) OIS + GBR; (c) TIS; (d) OIS.

No significant interaction was observed between membrane use and implant surface for any variable. Histomorphometric images of the groups are shown in Figure 4.

Regarding membrane use, the height of the newly formed bone was significantly greater in the membrane-treated group than in the non-membrane-treated group, irrespective of implant surface type (p = 0.037). Implants treated with GBR exhibited both greater thickness and height of newly formed bone at the base of the defect (TDb) than oxidized implants without GBR (p = 0.004), but no significant differences were observed compared to turned implant surfaces. In the central region of the bone defect, oxidized implants showed greater bone formation than turned implants (p < 0.001) (Table).

**Table:** Histometric measurements of bone height and thickness (mm) at the base and central region of the defect.

Parameter	Oxidized surface (mean + SD)	Turned surface (mean + SD)	F	p-value*

HD				
With GBR	4.08 ± 1.67	4.37 ± 1.59		
Without GBR	1.89 ± 1.66	3.56 ± 1.80		
GBR			7.936	0.008
SURFACE			3.388	0.074
GBR*SUR			1.699	0.201
TDb				
With GBR	2.75 ± 1.12	2.27 ± 1.38		
Without GBR	0.93 ± 0.94	1.43 ± 0.81		
GBR			14.962	< 0.001
SURFACE			0.001	0.977
GBR*SUR			2.034	0.162
TDc				
With GBR	2.26 ± 1.02	1.87 ± 1.30		
Without GBR	0.30 ± 0.56	0.65 ± 0.59		
GBR			29.708	< 0.001
SURFACE			0.006	0.940
GBR*SUR			1.615	0.212

HD: height of the defect; TDb: thickness at the base of the defect; TDc: thickness in the central region of the defect; GBR: Guided Bone Regeneration. *Two-way ANOVA (p=0.05).

Pearson correlation^15^ revealed a strong positive association between the thickness and height of the newly formed bone at the base of the defect (r = 0.727; p < 0.001). A significant correlation was also observed between the thickness of newly formed bone at the base of the defect and its thickness in the central region (r = 0.932; p <0.001). Thus, greater bone thickness at the base of the defect was associated with increased bone height, and similarly, greater bone thickness at the base corresponded to increased bone thickness in the central region. No other correlations were statistically significant.

## Discussion

The present study demonstrated a positive correlation between the height and thickness of newly formed bone at both the base and central region of the surgically created dehiscence defect. It may be suggested that a greater residual thickness at the base of the defect facilitates the recruitment of host cells from the surrounding bone, thereby enhancing the healing process beneath the membrane.^6^ Spray et al.^15^ compared changes in vertical bone dimensions and thickness of the facial bone below the crest bone between implant placement and second-stage (uncovering) surgery in healed bone. Their findings suggested that vertical bone loss decreases as thickness increases. The authors reported the greatest amount of bone loss when facial bone thickness ranged from <1 to 1.4 mm, and identified a critical thickness of approximately 2 mm to minimize the risk of facial bone loss. Some evidence of bone gain was also observed when bone thickness ranged from 1.8 to 2 mm. Despite differences in study design and measurement methods, the present study found mean residual bone thicknesses at the base of the defect ranging from 0.93 to 1.43 mm in non-regenerated sites, and from 0.30 to 0.65 mm in the central region of the defect.

The findings of this study also demonstrated that buccal dehiscence defects measuring 5 mm in height and 4 mm in width were completely covered with new bone when treated with GBR, and exhibited osseointegration at the implant surface. The use of the membrane prevents direct contact between the bone defect and soft tissues, thereby facilitating bone regeneration,^7,8^ since it may protect against the loss of growth factors from host cells.^16^ The minimal dimensions of bone defects that can heal spontaneously or require GBR have not been clearly established. Zitzmann et al.^17^ evaluated the use of an e-PTFE membrane and bone graft in the treatment of bone defects around implants. Their study showed that changes in marginal bone levels were greater in regenerated implants than in those without GBR. The authors concluded that regeneration is recommended for defects exceeding 2 mm in the vertical dimension.

The present study demonstrated that bone formation increased when non-resorbable e-PTFE membranes were used in animals under well-controlled conditions. A recent systematic review evaluating 15 clinical trials found that e-PTFE membranes achieved better outcomes in dehiscences up to 5 mm compared to polylactic and polyglycolic acid (PLGA) membranes.^18^ However, no significant differences were observed when ePTFE membranes were compared with resorbable collagen membranes. Schneider et al.^8^ reported that e-PTFE membranes promoted greater vertical bone gain compared to the PLGA membrane, likely due to the potential partial collapse of PLGA membranes after application and their early resorption before complete bone formation. Despite the current discontinuation of e-PTFE membranes, they and other non-resorbable membranes remain widely used. Thus, considering the rigidity of the non-resorbable e-PTFE membrane and its ability to maintain space without grafting, the authors believe that evaluating vertical and horizontal bone in dehiscence defects is particularly relevant in this study. However, the requirement for a second surgery to remove the membrane after implant placement and the risk of membrane exposure and contamination remain notable drawbacks of non-resorbable membranes (e-PTFE, d-PTFE, and titanium mesh) in certain clinical situations. Clinicians should carefully assess patient-specific needs when recommending the type of membrane to be used for the successful outcome of bone regeneration.

Chiapasco et al.^5^ also reported that GBR is a reliable approach for treating bone fenestrations and dehiscences without requiring graft materials. Jung et al.^1^ found that patients with implants placed in small, non-contained buccal bone dehiscences (≤ 5 mm) that were left to heal spontaneously exhibited high implant survival rates and healthy peri-implant tissues. Waller et al.^4^ demonstrated that smaller vertical dehiscences (≤ 5 mm) were observed in implants treated with GBR compared to those without GBR. Additionally, implants with membranes exhibited greater vestibular bone thickness and a slight vertical vestibular gain, findings that align with the present study. However, implants with small, non-contained buccal bone dehiscences left to heal spontaneously also exhibited high implant survival rates and healthy peri-implant tissues at 7.5 years of follow-up.

A previous clinical study demonstrated that, in absence of a membrane, the most coronal region of the implant is composed solely of mucosa rather than both bone and mucosa, which may lead to recessions over time and thread exposure. In patients with poor hygiene, exposed threads can accumulate biofilm and contribute to inflammation in the peri-implant area.^1^ In contrast, Jung et al.^1^ reported greater vertical bone loss at the buccal aspect within the first six months after implant placement; however, GBR procedures enhanced the stability of the buccal bone in implants with buccal bone dehiscence defects. In the present study, bone regeneration was observed at both the base and central portion of the defect when a membrane was used, as well as in the vertical direction. As a result, the submerged implant achieved greater bone anchorage up to the top of the threads, preventing the formation of a coronal region consisting only of soft tissue. This outcome may reduce the likelihood of recessions and potentially minimize inflammatory processes.

Modifications to the microtopography and nanotopography of dental implants have been introduced to enhance osseointegration.^19^ Hussain et al.^20^ reported that implant surface characteristics contribute to osseointegrative properties, demonstrating a positive correlation between the surface roughness and the strength of the bone-implant interface. Enhancing biocompatibility and cell viability requires adjustments to the implant surface, since osteoblastic activity is greater on rough (treated/oxidized) surfaces than on smooth (untreated/turned) surfaces,^9^ and bone-implant contact is greater in implants with rougher surfaces.^19^ However, the results of the present study showed no improvement in bone height or thickness associated with implant surface treatment during healing. Bone formation in turned and oxidized implants at the base of the defect was comparable to bone formation in the upper portion and central region of the defect.

The reduced vascular supply in the connective tissue adjacent to the implant^21,22^ may interfere with the healing process, bone formation in dehiscence defects, and even the defense system.^21^ When GBR is applied to non-space-making defects, such as dehiscence defects, with or without bone grafts, it creates an isolated space that allows the blood clot to serve as a matrix for bone healing.^23^In large residual buccal defects, increased new bone formation may also occur at the base of the defect due to the lateral aspects of the defect being protected under the membrane.^7^

In clinical practice, managing bone defects requires evidence-based protocols that consider both treatment efficacy and the esthetic outcome of implant rehabilitation. When the implant surface is left exposed, whether during alveolar preparation for immediate implant placement in extraction sockets at healed sites, or in submerged or non-submerged implant situations, complications may arise. Such complications include soft tissue recession and implant exposure to the oral environment, which may compromise the success of implant rehabilitation.

Thus, the results presented in this study should be interpreted with caution due to the small sample size, a common limitation in animal studies. Unlike peri-implantitis defects,^21^ the bone at the base of surgically created experimental defects is likely to be thicker, whereas the bone in clinically occurring dehiscence defects tends to be thinner and narrower. In such cases, a membrane may remain in contact with the implant surface without sufficient space for bone formation, or with only a minimal amount of bone. In contrast, when the bone at the base of the dehiscence is thick, space may be present due to the anatomy of the defect. However, an experimentally created defect is unlikely to occur in routine implant placement surgeries. Additionally, since all the implants in this study were submerged, no measurements of mucosa width, soft tissue thickness or soft tissue level were conducted to assess the influence of soft tissue on bone healing. The ePTFE membrane used in this study has become less common in recent years, primarily due to the need for a second surgery for removal and the successful outcomes observed with resorbable membranes. However, ePTFE remains the gold standard among non-resorbable membranes for vertical GBR, given its biocompatibility, space-maintaining properties, and ease of handling.^24,25^ This membrane serves as a cell-occlusive barrier and is a chemically stable, biologically inert polymer that promotes undisturbed healing. It also resists enzymatic and microbiological degradation and does not trigger immunological reactions.^24-26^ No imaging analyses were performed in this study to provide additional information on bone formation. For instance, cone beam computed tomography (CBCT) could have provided further peri-implant bone measurements.^27^ Another limitation is that mechanical stability under loading was not assessed, despite the fact that mechanical loads on implants can stimulate bone remodeling at the bone-implant interface.^28^

Despite the limitations of this study, it provides valuable insights into bone formation, particularly regarding thickness at the base and central portion of the defect, as well as defect height. These improvements appear to be more strongly associated with GBR using non-resorbable ePTFE membranes than with implant surface type. Further long-term clinical studies are needed to confirm these findings in larger populations with diverse implant installation scenarios and different implant designs. Additionally, preclinical studies should be conducted to better understand the behavior of hard and soft tissues, and to support the development of a standardized protocol for treating bone dehiscences around implants, ultimately ensuring the long-term success of implant rehabilitations.

## Conclusion

Based on the findings of this study, vertical and horizontal bone formation around implants with 5 mm dehiscence defects were correlated, with horizontal bone formation significantly influencing vertical bone growth. While guided bone regeneration with non-resorbable ePTFE membranes enhanced bone formation regardless of implant surface type (oxidized or turned), these results should be interpreted in the context of current clinical practice. Although ePTFE membranes are less commonly used today, the findings suggest that barrier membrane techniques remain important for optimizing bone formation around implants with dehiscence defects. Future studies should explore whether contemporary approaches and membrane materials can achieve comparable outcomes, potentially offering a more clinically practical solution for guided bone regeneration in implant therapy.

## Data Availability

Data is available on demand from the reviewers.
